# Effects of Electric Current on the Mechanical Properties of Cu/Nb Multilayer Composites by Accumulative Roll Bonding

**DOI:** 10.3390/ma18092109

**Published:** 2025-05-04

**Authors:** Chenghang Ni, Chaogang Ding, Fanghui Wang, Hushan Li, Qiang Zhu, Debin Shan

**Affiliations:** 1 CGN-HIT Advanced Nuclear and New Energy Research Institute, Harbin Institute of Technology, Harbin 150001, China; 17280310612@163.com; 2 National Key Laboratory for Precision Hot Forming, Harbin Institute of Technology, Harbin 150001, China; wangfanghui1014@163.com (F.W.); hushan1124@163.com (H.L.); shandb@hit.edu.cn (D.S.); 3 Key Laboratory of Micro-Systems and Micro-Structures Manufacturing of Ministry of Education, Harbin Institute of Technology, Harbin 150001, China; 4 School of Materials Science and Engineering, Harbin Institute of Technology at Weihai, Weihai 264209, China

**Keywords:** Cu/Nb multilayer composite, electrically assisted tension, strain distribution, mechanical property, fracture behavior

## Abstract

Cu/Nb multilayer composites with a continuous layered structure were fabricated using accumulative roll bonding (ARB). The effects of Joule heating on the mechanical properties and fracture behavior of these composites under electrically assisted tension (EAT) at different current densities were investigated. It is observed that the ultimate tensile strength (UTS) exhibits a progressive decline with the increasing current density. When the current density reaches 120 A/mm^2^, the UTS decreases by 68.9 MPa, and this decline tends to saturate at high current densities. Furthermore, the elongation (EL) displays significant enhancement at current densities of 40 A/mm^2^ and 80 A/mm^2^, particularly reaching a maximum improvement of 42.1% at 80 A/mm^2^ when compared with room temperature (RT). The fracture mode observed during the EAT process is consistent with that at RT, which remains a ductile fracture.

## 1. Introduction

Heterostructured materials represent a novel class of materials characterized by heterogeneous zones exhibiting significantly different mechanical or physical properties [1]. The synergistic effects arising from the interactive coupling between these heterogeneous zones lead to an overall performance that surpasses predictions based on the rule-of-mixtures [2,3]. This showcases unique performance advantages and broad application prospects, which has garnered considerable attention within the field of materials science research [4,5]. Among them, multilayer composites are a particularly promising type of heterostructured material achieved by the composite technology that enables the alternating arrangement of two or more metallic materials, typically consisting of alternating layers of various materials with strong interfacial bonding formed between these layers [6,7,8]. Research has demonstrated that such multilayer composites can significantly enhance many properties such as fracture toughness, fatigue behavior, impact behavior, wear, corrosion, and damping capacity, or provide improved formability or ductility for other brittle materials [7,9]. Currently, the fabrication of multilayer composites can be achieved using a “bottom-up” approach, which utilizes the physicochemical properties of the materials themselves to allow different atoms to alternately deposit onto a substrate under the influence of an electric or magnetic field, thereby assembling thin film materials with a nanolayered structure, among which the deposition technique is the most typical preparation process [10,11,12]. Unfortunately, most of these methods require complex processes and expensive equipment, making it challenging to produce multilayer composites on a large scale and in bulk, thereby limiting their industrial applications.

Accumulative roll bonding (ARB), a severe plastic deformation technique that accumulates strain through multiple rolling deformations, enables the repeated rolling of large-scale materials while maintaining their overall size. This method facilitates significant cumulative deformation, grain refinement, and continuous production [13,14,15]. ARB has been widely utilized by numerous researchers in the preparation of multilayer composites, including Cu/Ti [16,17], Cu/Al [18,19], Cu/Ni [20,21], Ti/Al [22,23], Al/Mg/Al [24,25], and Cu/Nb [26,27]. Jiang et al. [16] fabricated Cu/Ti multilayer composites through the ARB and the Cu and Ti layers are bonded well during the ARB processing. In addition, as the number of ARB cycles increased, the Ti layers began to neck, fracture, and even segregate within the Cu matrix. Eizadjou et al. [18] prepared Al/Cu multilayer composites using ARB, and found a higher strength enhancement compared to the tensile strength of Al/Al multilayered strips produced by ARB. Tayyebi et al. [20] fabricated Cu/Ni multilayer composites through ARB. Their findings indicated that increasing strain during ARB cycles led to increases in both the strength and elongation of these composites. Notably, Cu/Nb multilayer composites, with their high-density interfaces that not only significantly impede dislocation motion to enhance strength and hardness but also effectively absorb and facilitate the annihilation of radiation defects, exhibit outstanding resistance to radiation damage, making them promising candidates for high-temperature and radiation-rich environments [27,28,29,30]. This provides innovative solutions for the development of materials designed to withstand challenging operational conditions. However, the suppression of dislocation storage in multilayer composites leads to increased deformation resistance and poorer formability at room temperature (RT), making it difficult for the loads applied by molds in traditional forming techniques to meet the stringent requirements for dimensional accuracy, surface quality, and microscopic defect control in some precision components, thus becoming a bottleneck that restricts their widespread application [31,32]. Therefore, developing precision forming and manufacturing techniques for multilayer composite materials will be the key to breaking through this bottleneck.

In recent years, electrically assisted forming (EAF) has been applied to improve the formability of difficult-to-form materials. Research shows that applying electric current to the blank during the plastic forming process can effectively improve the forming performance of metal sheets, reduce forming loads, and enhance the quality of micro-formed components [33,34,35,36]. Salandro et al. [37] designed a current-assisted micro-forming mechanical property testing device, revealing that the micro-deformation flow stress of materials significantly decreased under electrical excitation, demonstrating notable electroplasticity. Minor et al. [38] investigated the electroplasticity in Ti-Al alloys and found that electrical current enhanced dislocation cross-slip and twinning, substantially improving the material’s plastic deformation capability. Kim et al. [39] discovered that electrical current induced significant flow stress softening effects and dramatically increases material elongation. In summary, EAF offers unique advantages such as low forming loads, high efficiency, low costs, and high precision of formed components. Therefore, EAF has significant advantages in the precise micro-forming manufacturing of high-performance micro components. In particular, electrically assisted tension (EAT) has been widely employed to investigate mechanical properties and optimize the forming processes of various lightweight alloy systems, leading to breakthrough advancements in titanium alloys [40,41,42], magnesium alloys [43,44,45], and aluminum alloys [46,47].

However, current research on the electroplasticity in sheet materials remains predominantly focused on single-layer metallic materials, with limited studies on the influence of EAF on the forming properties of multilayer composites. This gap is particularly pronounced in the field of Cu/Nb multilayer composites, leading to insufficient theoretical guidance for the design and optimization of EAF for multilayer composites, which has hindered the development of forming processes for multilayer composites. Therefore, this study successfully fabricated Cu/Nb multilayer composites with continuous layered structures through the multi-pass ARB by carefully adjusting the rolling parameters. Then, this study further investigated the temperature variation due to Joule heating in Cu/Nb multilayer composites under different current densities. Moreover, through the EAT process, the influence of current parameters on mechanical properties and fracture behavior of Cu/Nb multilayer composites during micro-tensile tests was studied. Ultimately, this study provides both a theoretical foundation and experimental support for elucidating the mechanisms of EAF in the forming process of multilayer metal composite materials.

## 2. Materials and Methods

### 2.1. Sample Preparation

**Figure 1:**
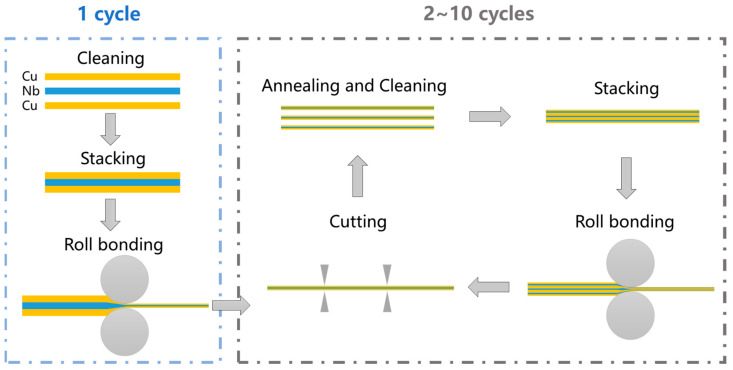
Schematic of the ARB process of a Cu/Nb multilayer composite, yellow represents Cu, blue represents Nb, and circles represent rollers.

Commercially pure Cu (99.9 wt%) and pure Nb (99.9 wt%), measuring 50 mm × 60 mm × 1 mm were selected as the subjects of this research. The original Cu and Nb plates underwent annealing at 500 °C for 1 h and 1050 °C for 1.5 h, respectively, under an argon atmosphere. Figure 1 illustrates the schematic of the ARB process of a Cu/Nb multilayer composite. Firstly, the surface to be rolled was polished with a wire brush, followed by ultrasonic cleaning in acetone to eliminate the oxide layer and impurities on the surface of the plates, thereby enhancing the quality of the interface welding after rolling. Afterward, the Nb plate was sandwiched between two Cu plates, and rolled at room temperature using a dual roll mill. The rolling equipment used in the rolling process is a two-roll mill. The maximum available rolling force of this equipment is 200 kN. The material of the rolls is 9Cr2Mo, the surface hardness is approximately 60 HRC, the diameter of the rolls is 170 mm, the maximum rolling width is 185 mm, and the rotational speed is 12 rpm. Through the rolling process, approximately 78% of the deformation of the first rolling pass was achieved, and two materials were tightly bonded, resulting in the formation of a Cu/Nb multilayer composite structure. Subsequently, the rolled Cu/Nb multilayer composite plate was cut by the plate shears into three equal sections and insulated for 2 h at 600 °C under an argon atmosphere to equilibrate the strength differences between Cu and Nb. Following this, 10 cycles of grinding, superposition, rolling (achieved approximately 50% of the deformation), shearing (divided into two parts), and annealing were carried out, and more details were described in reference [28,48,49,50,51]. Ultimately, Cu/Nb multilayer composites fabricated using ARB with 10 cycles were successfully obtained.

### 2.2. Experimental Procedures

**Figure 2:**
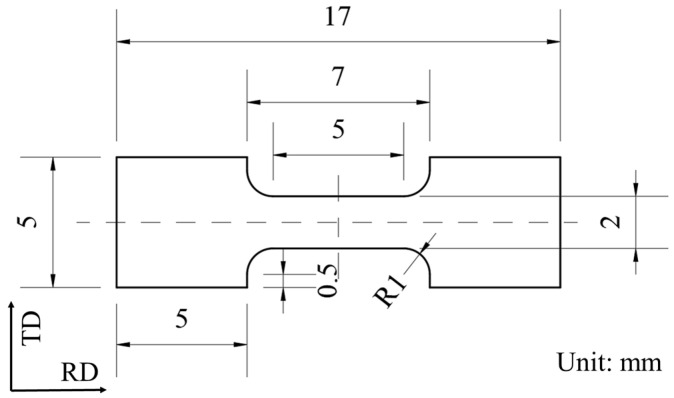
Tensile specimen size of Cu/Nb multilayer composites.

A dog bone tensile specimen with nominal dimensions of 5 mm × 2 mm was cut from a Cu/Nb multilayer composite plate using the electrical discharge machining method. The specific dimensions of the tensile specimen are shown in Figure 2. The surfaces and edges of the cut tensile specimen were polished to remove processing marks. Furthermore, pulse currents with current densities of 40 A/mm^2^, 80 A/mm^2^, and 120 A/mm^2^ were applied to the sample, with a pulse width of 0.004 s and a pulse period of 0.02 s. Once the sample reached its equilibrium temperature, it was stretched and continuously deformed until it fractured at a strain rate of 0.001 s^−1^. To ensure the repeatability and reliability of the experimental results, all tensile tests were repeated at least three times.

The EAT process was conducted on a self-developed current-assisted micro-tensile experimental platform within the research group. This platform integrates several key components: an electronic universal material testing machine (AG-X 50 KN, Shimadzu Corp., Kyoto, Japan), a pulse power supply (MicroStar CRSL-FP20-500, Dynatronix Inc., Amery, WI, USA), an infrared camera (FLIR T660, Wilsonville, OR, USA), a CCD camera, and a micro-tensile fixture. The system facilitates the load application, real-time monitoring, and control of current and temperature, as well as full-field strain analysis using digital image correlation (DIC). The DIC measurement system consists of a CCD camera (GS3-U3-15S5M-C, FLIR Systems Inc., Wilsonville, OR, USA), CCD macro-lens (Navitar 12X, NAVITAR Inc., Rochester, NY, USA), light source, image acquisition software (VIC-Snap, Version 8, Correlated Solutions Inc., Irmo, SC, USA), speckle tool, and image analysis software (VIC-2D, Version 6.02, Correlated Solutions Inc., Irmo, SC, USA). The morphology of the Cu/Nb multilayer composites along the rolling direction-normal plane and the tensile fracture was observed using scanning electron microscopy (SEM, ZEISS Gemini 560, Oberkochen, Germany), while the distribution of elements was analyzed through energy dispersive spectroscopy (EDS).

## 3. Results and Discussion

### 3.1. Microstructure

**Figure 3:**
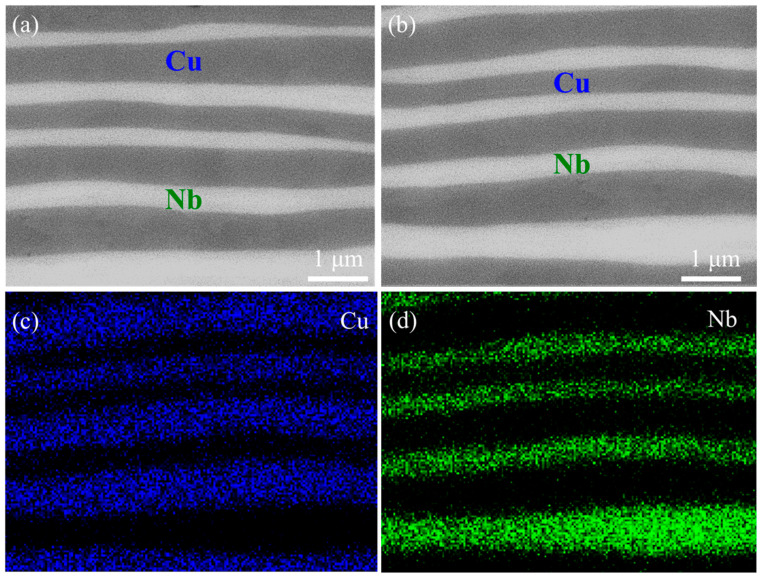
Microstructure of Cu/Nb multilayer composites: (**a**,**b**) SEM cross-section morphology; (**c**,**d**) EDS element distributions.

Figure 3 shows the microstructure of Cu/Nb multilayer composites after 10 cycles of ARB. The SEM cross-sectional morphology of the Cu/Nb multilayer composite along both the rolling direction and the normal plane is shown in Figure 3a,b. It is evident that the Cu/Nb multilayer composite maintains an intact laminar structure, with a well-defined interface devoid of cracks and voids, indicating strong interfacial bonding. Although wavy and irregular morphologies appeared at the interface, there are no signs of necking or fracture. Figure 3c,d depict the distribution of elements corresponding to Figure 3b, which further confirms that the Cu and Nb layers exhibit a regularly alternating arrangement, demonstrating the absence of detectable impurities throughout the layered structure. The elemental analysis further demonstrates that neither does a reaction occur between the Cu phase and the Nb phase nor are intermetallic compounds formed. These results have been confirmed in previous studies [28]. These microstructural characteristics suggest that Cu/Nb multilayer composites demonstrate superior deformation compatibility and structural stability during ARB [49].

### 3.2. Temperature Variation

**Figure 4:**
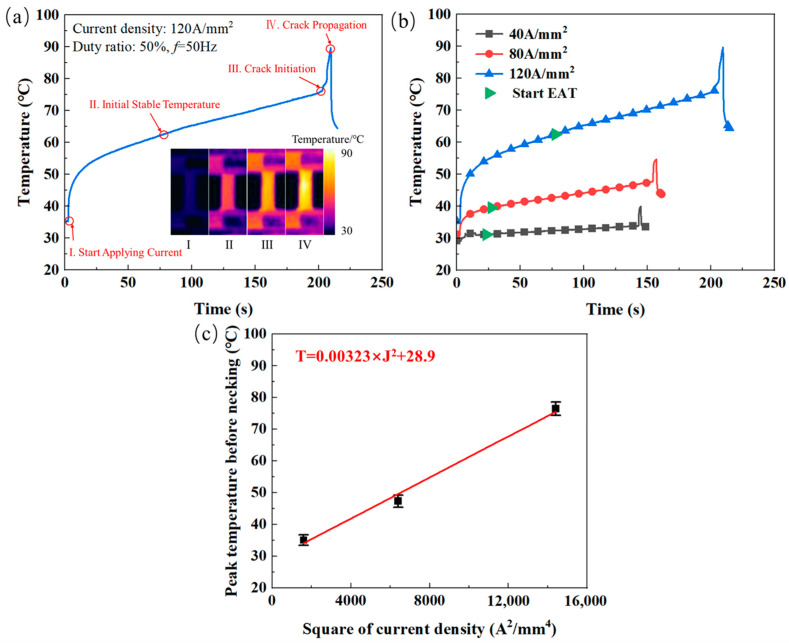
Temperature fields of Cu/Nb multilayer composites during the EAT process: (**a**) typical four stages of temperature field variation; (**b**) temperature variation with time and current density; (**c**) peak temperature of the samples before necking under different current densities.

Figure 4 exhibits four stages of temperature field evolution during the EAT of Cu/Nb multilayer composites, along with the corresponding temperature variations over time and current density. As depicted in Figure 4a, the temperature evolution during the EAT process can be segmented into four distinct stages, namely (I) temperature distributions before tension, (II) initial steady-state temperature, (III) crack initiation, and (IV) crack propagation stages, which is consistent with previous research findings [52]. When the electric current is applied to the specimen, the temperature rapidly rises from RT (Stage I). Once the Joule heat generated by the electric current reaches dynamic equilibrium with thermal radiation and conduction losses, the temperature begins to stabilize (Stage II). Subsequently, the tensile test is started, with the initiation times under different current densities conditions clearly annotated in Figure 4b. As the stretching process progresses, the cross-sectional area of the specimen gradually decreases, leading to an increase in current density and a corresponding gradual rise in temperature (Stage III). During the necking phase, the sharp reduction in the cross-sectional area causes a sharp increase in temperature until it reaches the peak temperature, ultimately resulting in tensile fracture (Stage IV). As the early study reported [53], the area with defects has local high temperatures, which can be explained as the local Joule heat effect. This suggests that in the wavy and irregular morphologies regions of the microstructure mentioned earlier, the local Joule heating effect may occur, resulting in cumulative thermal effects in these areas. During the necking stage, the rapid temperature increase in the necking region is closely associated with crack initiation. Meanwhile, the reduction in the cross-sectional area in the necking region leads to a sharp increase in the actual current density, resulting in the local Joule thermal effect inducing transient thermal effects in this area, and a previous report also had the same finding [52]. Figure 4c shows the peak temperature of the samples before necking under different current densities, which demonstrates that the maximum temperature during the EAT process rises with increasing current density. At current densities of 40 A/mm^2^, 80 A/mm^2^, and 120 A/mm^2^, the peak temperatures before the onset of necking reach 35.1 °C, 47.3 °C, and 76.5 °C, respectively. It also shows that the square of the current density is proportional to the peak temperature before necking, consistent with Joule’s Law, which is the same as to the early research [42]. Joule’s Law states that the heat generated per unit volume is proportional to the square of the current density ($J^{2}$) and resistivity. Under steady-state conditions, the temperature rise (ΔT) is proportional to $J^{2}$:

(1) $$\Delta T=k\cdot J^{2},$$

where k is a proportionality constant incorporating material properties (e.g., resistivity, thermal conductivity) and experimental geometry [42]. Since the material properties remained constant, the relationship between the peak temperature before necking and the current density could be derived through data fitting and is presented in Figure 4c.

### 3.3. Local Strain Evolution

**Figure 5:**
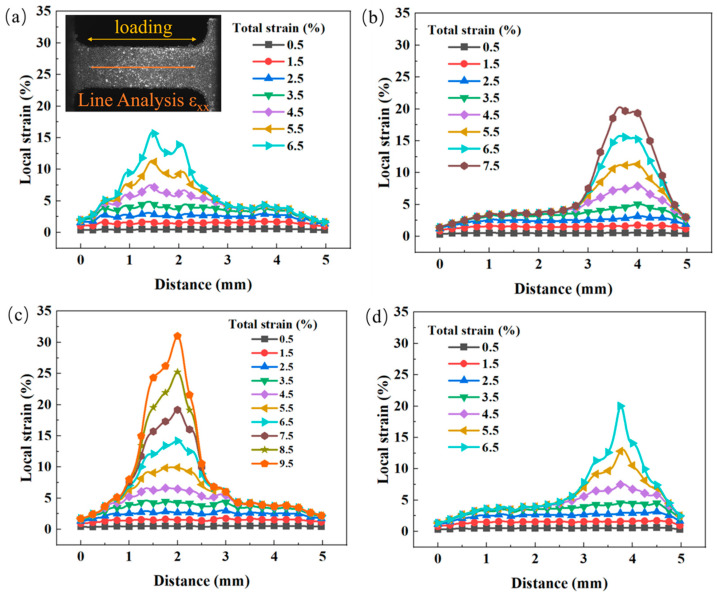
Strain distribution along the tensile direction of Cu/Nb multilayer composites at different strain stages under different current densities: (**a**) RT; (**b**) 40 A/mm^2^; (**c**) 80 A/mm^2^; (**d**) 120 A/mm^2^.

Figure 5 shows the local strain distribution ε_xx_ along the tensile direction in Cu/Nb multilayer composites subjected to different current densities during tensile deformation. As depicted in Figure 5a, a straight line was drawn along the stretching direction, serving as the path for local strain measurement, and the other figures are consistent with it. When the total strain is below 1.5%, the local strain at various positions on the specimen increases at a nearly consistent rate, indicating that no strain concentration occurs in the material. However, as the total strain increases to 2.5%, the local strain in the central region becomes significantly higher than that in the lateral regions, marking the onset of localized strain concentration. When the total strain reaches 4.5%, the local strain curve exhibits a peak value, confirming the formation of strain concentration. As shown in Figure 5, at low levels of total strain, the local strain within the material demonstrates similar patterns of variation. With further increases in total strain, the local strain in the lateral regions stabilizes while that in the central region continues to rise. Upon reaching a total strain of 6.5%, the strain concentration becomes more pronounced, especially under the condition of 120 A/mm^2^. As shown in Figure 5d, there is a prominent convex region appears in the local strain curve when the total strain is 6.5%, indicating a substantial strain concentration in this area. In contrast, Figure 5c shows that the bulge in the local strain curve is relatively minimal at 80 A/mm^2^ under the same total strain, suggesting that strain concentration is weakest at this current density. As the total strain continues to increase, there is no subsequent local strain curve due to sample fracture in Figure 5a,d, while the local strain continues to increase until fracture occurs in Figure 5b,c. The strain concentration phenomenon can be attributed to two main mechanisms: On the one hand, the severe plastic deformation induced by the ARB process significantly weakens the material’s work hardening capacity, thereby reducing its uniform deformation capability and predisposing it to localized strain concentration [50]. On the other hand, during the EAT process, due to the initial local deformation, the local cross-sectional area of the specimen decreases, resulting in an increase in the actual current density in this area and a rise in the local temperature, which further intensifies the strain concentration [54]. To further compare the degree of local strain concentration, in this paper, the degree of local strain concentration is evaluated by the damage factor D:

(2) $$D={\bar{\varepsilon }}_{Max}/{\bar{\varepsilon }}_{Min},$$

where ${\bar{\varepsilon }}_{Max}$ is the average local strain in the strain concentration area, and ${\bar{\varepsilon }}_{Min}$ is the average local strain in the non-strain concentration area [55]. When the total strain reached 6.5%, the damage factors at RT, 40 A/mm^2^, 80 A/mm^2^, and 120 A/mm^2^ were calculated to be 4.58, 4.75, 3.90, and 5.47, respectively. These results suggest that during the EAT process, the strain concentration was minimal at 80 A/mm^2^ and maximal at 120 A/mm^2^.

**Figure 6:**
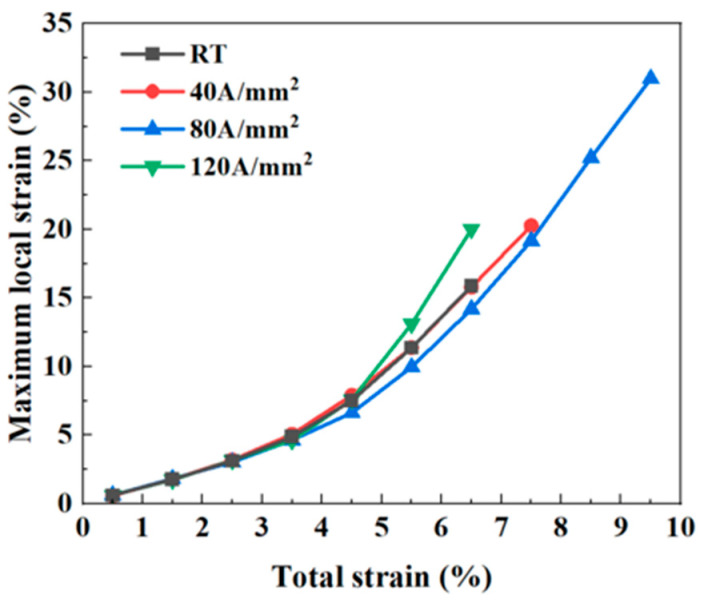
The maximum concentrated strain ε_max_ along the tensile direction of the samples under different current densities.

Figure 6 presents the curves of maximum local strain along the tensile direction as a function of total strain, which further validates this trend. At relatively low total strain levels, the maximum local strain increases linearly with the total strain. However, as the total strain increases, the growth curve of the maximum local strain begins to deviate upward. The curves corresponding to RT and a current density of 40 A/mm^2^ largely overlap, indicating minimal differences in maximum local strain between these two conditions. Notably, at a total strain of 6.5%, the curve for a current density of 120 A/mm^2^ exhibits a significant increase, with the maximum local strain reaching as high as 20%. This substantial rise is attributed to pronounced strain concentration within the material under this condition. In contrast, at a current density of 80 A/mm^2^, the maximum local strain only reaches 14.2%, which is notably lower than that observed in other conditions. This reduced value is primarily due to the relatively weak strain concentration present at 80 A/mm^2^.

**Figure 7:**
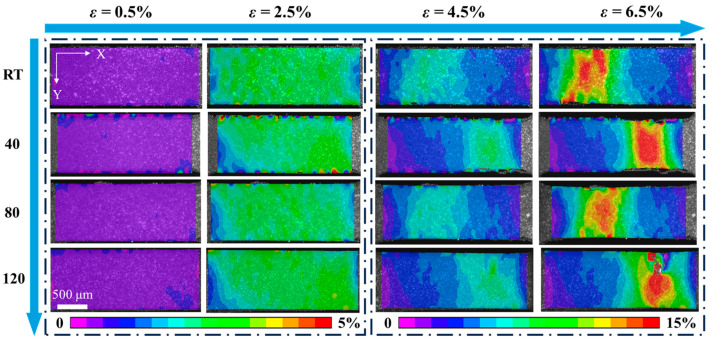
Local strain evolution of Cu/Nb multilayer composites at different current densities.

Figure 7 displays the local strain distribution under different current densities, with the x direction aligned parallel to the tensile direction. The resolution of the camera can reach 1384 × 1036. Using the VIC-2D software program, full-field strain distributions on the tensile specimen’s surface were successfully obtained via a DIC analysis of the specimen’s gauge section, captured with a camera resolution reaching 1384 × 1036 pixels. When the total strain is 0.5% and 2.5%, the strain field is evenly distributed; when the total strain reaches 4.5%, the local strain gradient gradually appears. When the current density is 120 A/mm^2^, there is a large degree of strain concentration, and when the current density is 80 A/mm^2^, the strain concentration is the least. The results show that a low current density helps to reduce the strain concentration, while a high current density intensifies the strain concentration due to the temperature concentration [46]. Based on this characteristic, in practical applications, for complex components formed with Cu/Nb multilayer composites, based on the coupling characteristics of the temperature field and the strain field, infrared thermal imaging technology can be used to monitor the surface temperature distribution of the components in real time, and the internal strain distribution characteristics can be retrieved through the established temperature–strain conversion model. When abnormal local temperature increase areas are detected, the evolution trend of the strain concentration area can be predicted in advance, and gradient transition layers or additive reinforcement structures can be set for key parts to effectively avoid the risk of failure.

### 3.4. Mechanical Properties

**Figure 8:**
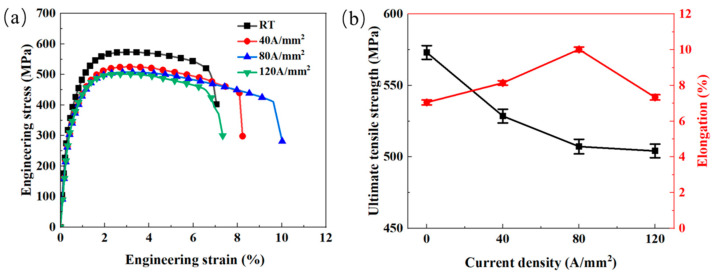
Mechanical properties of Cu/Nb multilayer composites at different current densities: (**a**) engineering stress–strain curves under different current densities; (**b**) variations in UTS and EL with current density.

Figure 8a indicates the engineering stress–strain curves of Cu/Nb multilayer composites under different current densities. It is observed that these curves nearly overlap at low strains. However, as the strain increases, the flow stress during the EAT process becomes significantly lower than that observed at RT. This phenomenon is mainly caused by enhanced Joule heating during the EAT process, leading to elevated temperatures and a subsequent decrease in flow stress. Figure 8b presents the variations in ultimate tensile strength (UTS) and elongation (EL) with current density. The experimental results reveal that the UTS decreases with an increasing current density. For instance, the UTS at RT is 573.1 MPa, and when the current density reaches to 120 A/mm^2^, the UTS reduces to 504.2 MPa, which decreases by 68.9 MPa. Specifically, significant reductions of 8.4%, 11.4%, and 12.4% are observed at 40 A/mm^2^, 80 A/mm^2^, and 120 A/mm^2^, respectively. On the one hand, this trend is attributed to the electroplasticity during electrical stimulation: the introduction of electric current reduces the resistance to dislocation motion, and promotes dislocation migration, thereby leading to a decrease in the flow stress [36]. On the other hand, according to the early research [56], the application of electric current during plastic deformation induces a “mosaic temperature field” in metallic materials, characterized by localized heating at micro-defects such as grain boundaries, which inevitably causes localized softening and decrease in stress. Notably, although the temperature at 120 A/mm^2^ was substantially higher than at 80 A/mm^2^, the difference in the UTS between these two conditions remained minimal. This result indicates that the influence of electroplasticity on UTS tends to saturate at high current densities [57]. The EL demonstrates a positive correlation with current density below 80 A/mm^2^. At RT, the EL measures 7.05%, while the application of 40 A/mm^2^ and 80 A/mm^2^ current densities elevates this value to 8.15% and 10.02%, corresponding to 15.6% and 42.1% increases. This phenomenon originates from that the pulse current reduces the strain concentration, thereby enhancing the EL [46]. However, at higher current densities, since the actual current density rises as the cross-sectional area of the sample decreases during deformation, it can lead to a sharp temperature increase in localized regions following crack initiation. As a result, the Joule heat temperature distribution becomes uneven, leading to local temperature rise, uneven deformation intensification, and premature fracture [52,54].

**Figure 9:**
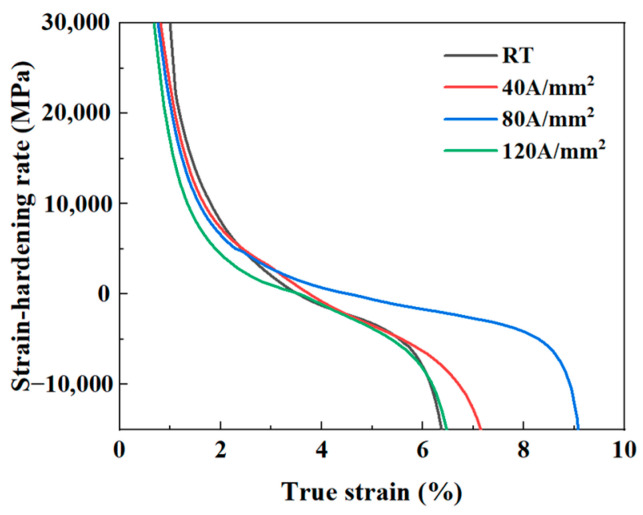
Strain-hardening rate curves of Cu/Nb multilayer composites at different current densities as a function of true stain.

As shown in Figure 9, the relationship between the strain hardening rate (SHR) and true strain for Cu/Nb multilayer composites at different current densities is derived from the true stress–strain curves. Throughout the deformation process, the SHR exhibits a downward trend. In the initial deformation stage (early true strain), the material primarily undergoes elastic deformation, and as the strain increases, the SHR decreases sharply. As deformation progresses further, the material gradually transitions into the plastic deformation stage. At this point, the rate of decrease in SHR gradually slows down, ultimately entering a gently declining plastic deformation stage, until a sharp decline occurs again. This trend is consistent with other studies [58,59]. By comparing the SHR trends under different current density conditions, it can be observed that at 120 A/mm^2^, the SHR curve first enters the gently declining stage, followed by 80 A/mm^2^, and lastly by RT and 40 A/mm^2^. This result indicates that the material enters the plastic deformation stage earlier under the conditions of 120 A/mm^2^ and 80 A/mm^2^, likely due to the Joule heating effect reducing the flow stress, which facilitates an earlier transition to plastic deformation. This phenomenon is closely related to the temperature changes illustrated in Figure 3. Notably, at a current density of 80 A/mm^2^, the duration of the gently declining plastic deformation stage is the longest, suggesting that the material exhibits the best plasticity at this current density, which is consistent with the optimal elongation results at 80 A/mm^2^ shown in Figure 7. However, when the current density reaches 120 A/mm^2^, although the material enters the plastic deformation stage earlier, the duration of this stage is relatively short. In the later stages of the SHR curve, there is a premature sharp decline in the material, primarily due to the Joule heating effect causing a significant increase in sample temperature, which enhances the thermal softening effect. This effect dominates the material’s response mechanism, rapidly diminishing the strain hardening capacity and ultimately leading to the premature termination of the plastic deformation stage [46].

### 3.5. Fracture Morphologies

**Figure 10:**
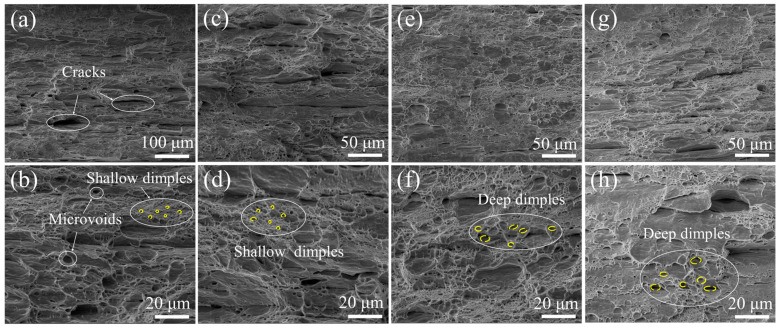
Fracture morphologies of Cu/Nb multilayer composites during the EAT process with different current densities: (**a**,**b**) RT; (**c**,**d**) 40 A/mm^2^; (**e**,**f**) 80 A/mm^2^; (**g**,**h**) 120 A/mm^2^.

Figure 10 presents the tensile fracture morphologies of Cu/Nb multilayer composites under different current densities. At RT, as shown in Figure 10a,b, the fracture surface of these composites exhibits numerous cracks, along with a significant number of shallow dimples and microvoids. According to the earlier report [60,61,62], it can be inferred that the fracture mode at RT is a ductile fracture. At the small current density of 20 A/mm^2^, there is no obvious change in fracture morphology compared with RT. When current density reaches 40 A/mm^2^ and 80 A/mm^2^, there are evidently a number of larger and deeper dimples that can be observed in Figure 10f,h. To further investigate the effect of current on the size of dimples at tensile fractures, the Image-Pro Plus software program was used to measure the average diameter of dimples marked in yellow within the regions in the figure. The average diameters of the dimples at RT and current densities of 40 A/mm^2^, 80 A/mm^2^, and 120 A/mm^2^ were 2.13 μm, 2.49 μm, 4.82 μm, and 5.22 μm, respectively. Compared with RT, the average diameters of the dimples increase by 17%, 126%, and 145%, respectively, at current densities of 40 A/mm^2^, 80 A/mm^2^, and 120 A/mm^2^. The results show that the current increases the size and depth of the dimples. Based on previous reports [23,62,63], this might be attributed to the fact that the application of current can suppress crack initiation or enhance crack propagation resistance, thereby enhance the ductility. This result further verifies that EL increases obviously when the current density reaches 80 A/mm^2^. Moreover, the fracture mode of Cu/Nb multilayer composites remains a ductile fracture even under electrified conditions, demonstrating that the application of electric current does not alter the fundamental fracture behavior of these composites.

## 4. Conclusions

In this paper, Cu/Nb multilayer composites were fabricated using ARB. Subsequently, the variation in Joule heating temperature under different current densities was investigated. Additionally, the influence of current parameters on mechanical properties and fracture behavior of Cu/Nb multilayer composites was analyzed.

(1) The peak temperature of Cu/Nb multilayer composites increases with the increase in current density, and the relationship between the maximum temperature before necking and the current density satisfies Joule’s heat law. Additionally, the strain concentration at a high current density is caused by the temperature increase and uneven temperature distribution.
(2) A pulsed current reduces the UTS of Cu/Nb multilayer composites, and this effect tends to saturate at high current densities. Compared with the EL at RT, the EL is significantly improved when the current density is 40 A/mm^2^ and 80 A/mm^2^, but without significant change at 120 A/mm^2^, which is attributed to electric current reduces the strain concentration and enhance the ductility at low current density.
(3) Fracture morphologies show that the current increases the size and depth of the dimples. Furthermore, during the EAT process, the fracture mode of the Cu/Nb multilayer composite is a ductile fracture, and the application of current does not change the fracture mode of the Cu/Nb multilayer composite.

## Data Availability

The original contributions presented in the study are included in the article; further inquiries can be directed to the corresponding authors.
